# Association Between Obesity and Tinnitus Among Adults in the United States: A Cross-Sectional Analysis of the National Health and Nutrition Examination Survey 2015-2018

**DOI:** 10.7759/cureus.95973

**Published:** 2025-11-02

**Authors:** Ashir Ahtsham, Muhammad Faizan, Salmana Moneeb, Fatima Naeem, Muhammad Sabieh Khalid, Muhammad Muneeb Alrashid, Nauman Tauqeer, Vaneeza Arshad, Clifton M Chow

**Affiliations:** 1 Department of Internal Medicine, Lahore General Hospital, Lahore, PAK; 2 Department of Internal Medicine, Faisalabad Teaching Hospital, Faisalabad, PAK; 3 Department of Obstetrics and Gynecology, Mayo Hospital, Lahore, PAK; 4 Department of Internal Medicine, Mayo Hospital, Lahore, PAK; 5 Department of Internal Medicine, Shaikh Zayed Hospital, Lahore, PAK; 6 Department of Pathology, Islam Medical and Dental College, Sialkot, PAK; 7 Department of Physiology, Islam Medical and Dental College, Sialkot, PAK; 8 Department of Endocrinology, Shalamar Hospital, Lahore, PAK; 9 Department of Psychiatry, Harvard Medical School, Boston, USA

**Keywords:** logistic regression, nhanes, obesity, tinnitus, united states population

## Abstract

Introduction

Obesity, a growing concern worldwide, may be associated with the occurrence of tinnitus. Studies suggest that obese populations are at a greater risk of development or progression of tinnitus; however, the association remains underexplored. This study aimed to determine the effect of obesity on the prevalence of tinnitus in the US population.

Methodology

This cross-sectional analysis used de-identified data from the National Health and Nutrition Examination Survey (NHANES) 2015-2016 and 2017-2018 cycles, which included 5,452 individuals in the final analytic sample. Multivariate logistic regression was used to determine the association between obesity and tinnitus.

Results

A total of 5,452 adults aged ≥ 20 years from NHANES 2015-2018 were analyzed. Tinnitus was reported by 863 (17.2%) participants. It was more common among older adults, males, and those who were obese or depressed. Prevalence was higher in obese adults (433, 20.3%) as compared to non-obese adults (430, 15.0%) (*P* = 0.007). In regression analyses, obesity was significantly associated with tinnitus in the unadjusted model (odds ratio (OR) = 1.44; 95% confidence interval (CI): 1.11-1.87) and remained significant after adjusting for demographic and other covariates (OR = 1.41; 95% CI: 1.01-1.98).

Conclusions

The study concluded that obesity appears to be independently associated with an increased prevalence of tinnitus. The findings underscore the importance of considering weight management in people with tinnitus, and further research is needed to clarify this association.

## Introduction

Tinnitus is a common auditory symptom characterized by the perception of sound in the absence of an external auditory stimulus and affects a large segment of the population [1]. Approximately 10%-15% of the population is affected by tinnitus, explaining the widespread prevalence of the condition; however, underlying pathological pathways are still not fully understood [2]. These phantom sounds have an etiology involving multiple factors, usually including some irregularities in the central nervous system (CNS) and sensorineural hearing loss (SNHL) [3,4]. Several systemic conditions may also be associated with tinnitus, such as diabetes, cardiovascular disease, autoimmune factors, psychiatric issues, and obesity. It is considered that these conditions may result in chronic inflammation in the nervous system, metabolic irregularities, and oxidative stress, contributing to the development and progression of these abnormal sounds [5].

Obesity, a major global health concern, refers to excessive adiposity (body mass index (BMI) ≥ 30 kg/m²) and has been linked to major pathological conditions in humans, including cardiovascular disease and chronic systemic inflammation [6]. This exaggerated accumulation of adipose tissue, proinflammatory adipokines, and cytokines mediates abnormal functioning of endothelial tissue, increased insulin resistance, and a chronic inflammatory state in the body, which may lead to disrupted metabolic states, type 2 diabetes, atherosclerosis, and inflammation in multiple systems, including the nervous system [7]. These factors, when taken into account together, motivate interest in studying the relationship between tinnitus and obesity by combining insights from pathophysiology, demographic factors, epidemiology, and potential interventions. Despite growing evidence linking metabolic disorders to tinnitus, the direct association between obesity and tinnitus remains underexplored.

Although prior literature explains the role of metabolic and vascular factors underlying tinnitus in obese individuals [5], few have specifically studied obesity as an independent predictor of tinnitus. Using population-level estimates, this study aimed to understand whether obesity independently contributes to tinnitus prevalence beyond demographic and psychosocial covariates.

## Materials and methods

Study design and population

**Figure 1:**
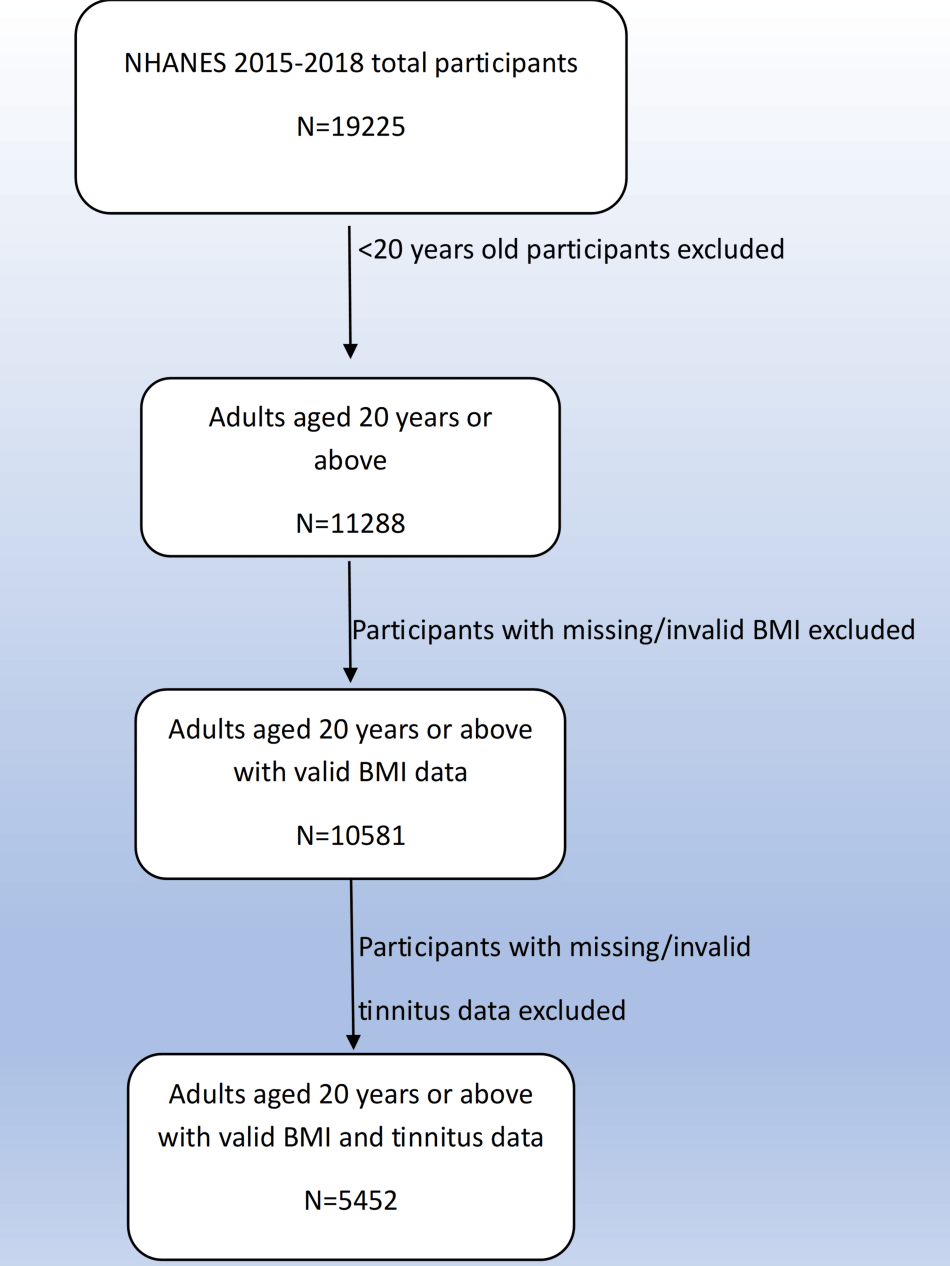
Flowchart of participant selection for the study sample. NHANES, National Health and Nutrition Examination Survey; BMI, body mass index

This cross-sectional study utilized publicly available data from the National Health and Nutrition Examination Survey (NHANES) 2015-2016 and 2017-2018 cycles, which is a nationally representative survey of the US population conducted by the National Center for Health Statistics (NCHS) [8]. The two aforementioned cycles were merged in accordance with NCHS analytic guidelines for enhanced statistical power. Participants from 2015-2018 were included in the study. All individuals aged less than 20 years and those having missing data for either BMI or tinnitus were excluded via listwise deletion to ensure analytic completeness. After applying these exclusions, a total of 5,452 participants were included in the final analytic sample (Figure 1). Sampling weights, strata, and primary sampling units (PSUs) were incorporated into all analyses to account for the complex survey design and to produce nationally representative estimates. Sample sizes varied across analyses due to missing data for specific variables.

Study variables

Obesity status of participants, based on BMI, was used as the independent variable for this study. Participants were classified as either non-obese (BMI < 30 kg/m²) or obese (BMI ≥ 30 kg/m²). Self-reported tinnitus, assessed via the NHANES audiometry questionnaire, was studied as the outcome of interest. Individuals who reported having experienced ringing, buzzing, or other noises in their ears for five minutes or more in the absence of an external sound were categorized as having tinnitus. Potential confounders, based on prior literature and their availability in the database, were used as covariates. These covariates included age (20-39 years, 40-59 years, and ≥ 60 years), sex (male or female), race/ethnicity (Hispanic, non-Hispanic White, non-Hispanic Black, and Asian/other), smoking (non-smoker, former smoker, or current smoker), sleep duration (< 7 hours daily or ≥ 7 hours daily), and depression, assessed using the Patient Health Questionnaire-9 (PHQ-9), with scores ≥ 10 classified as depression present [9,10].

Hearing loss data were not included in the analysis to preserve sample size and maintain consistency across the two survey cycles; however, this variable may mediate the relationship between obesity and tinnitus.

Statistical analysis

All statistical analyses were conducted using the complex samples module in IBM SPSS Statistics version 31.0.1.0 (IBM Corp., Armonk, NY), which accounts for the survey’s multistage design. Sampling weights, strata, and clusters were specified to account for NHANES’ complex survey design. A four-year examination weight (WTMEC4YR) was obtained by dividing the two-year MEC examination weight (WTMEC2YR) by two, as per the NHANES analytic recommendations for combining two survey cycles [11]. The participant characteristics, stratified by tinnitus status, were obtained using descriptive analyses. For bivariate analyses, Rao-Scott adjusted chi-square statistics were converted to *F* values and their corresponding *P*-values were reported. For logistic regression models, Adjusted Wald *F*-statistics were presented alongside *P*-values. A two-sided *P*-value of less than 0.05 was considered statistically significant.

The weighted prevalence of tinnitus was estimated by obesity status. Associations between obesity and tinnitus were evaluated using complex samples logistic regression models. In addition to a crude model comprising only obesity and tinnitus, demographically adjusted and fully adjusted models were also analyzed in order to determine the role of relevant covariates in the association between obesity and tinnitus. Results were reported as adjusted odds ratios (ORs) with 95% confidence intervals (CIs).

Portions of the results were drafted with the assistance of ChatGPT (OpenAI). However, the authors have reviewed and verified all details for accuracy and scientific integrity.

Ethical considerations

Our study involved secondary analysis of publicly available, de-identified data from NHANES that is approved by the NCHS Research Ethics Review Board and therefore did not require additional institutional review board (IRB) approval.

## Results

A total of 5,452 US adults from the 2015-2018 NHANES cycles were included in the analytic sample after excluding individuals younger than 20 years and those with missing data on BMI or tinnitus. Overall, tinnitus affected 863 (17.2%; 95% CI: 15.5-19.0) participants during 2015-2018. Its prevalence increased with age and was higher among men, non-Hispanic Whites, individuals with depression, and those who were obese. Multivariable modeling confirmed that obesity remained a significant predictor of tinnitus after controlling for potential covariates.

Participants with tinnitus tended to be older, more often male, and more likely to be obese or depressed compared with those without tinnitus. The prevalence of tinnitus increased markedly with age (*P* < 0.001), as we moved from adults aged 20-39 years (177, 10.5%) to those aged ≥ 60 years (416, 24.7%). Males (458, 18.7%) reported tinnitus slightly more often than females (405, 15.8%); however, the result was not statistically significant (*P* = 0.062). Across racial and ethnic groups (*P* < 0.001), non-Hispanic White (377, 19.5%) participants had the highest weighted prevalence of tinnitus, whereas non-Hispanic Black adults (143, 11.4%) had the lowest. Tinnitus prevalence was higher among obese adults (433, 20.3%) compared with non-obese adults (430, 15.0%) (*P* = 0.007). Although smoking status and sleep duration were not significantly associated with tinnitus (both *P* > 0.05), tinnitus was considerably more common among participants with depression (45, 26.7%) than among those without depression (335, 15.2%) (*P* = 0.009) (Table 1).

**Table 1:** Baseline characteristics of participants by tinnitus status. *P*-values are based on the Rao-Scott chi-square test converted to an *F*-statistic to account for the complex survey design of NHANES. The overall weighted prevalence of self-reported tinnitus among adults aged ≥ 20 years was 17.2% (*n* = 863; 95% CI: 15.5-19.0), derived from the total row of the complex samples crosstabs output. PHQ-9, Patient Health Questionnaire-9; BMI, body mass index; df1, numerator degrees of freedom; df2, denominator degrees of freedom; NHANES; National Health and Nutrition Examination Survey; CI, confidence interval

Variable	Category	Unweighted, *n*	No tinnitus, *n* (%)	Tinnitus, *n* (%)	Rao-Scott adjusted *F* (df1, df2)	*P*-value
Age group (years)	20-39	1,845	1,668 (89.5%)	177 (10.5%)	29.390 (1.953, 58.605)	< 0.001
	40-59	1,788	1,518 (82.1%)	270 (17.9%)		
	60+	1,819	1,403 (75.3%)	416 (24.7%)		
Sex	Male	2,614	2,156 (81.3%)	458 (18.7%)	3.757 (1, 30)	0.062
	Female	2,838	2,433 (84.2%)	405 (15.8%)		
Race/Ethnicity	Hispanic	1,573	1,311 (85.6%)	262 (14.4%)	9.328 (2.403, 72.094)	< 0.001
	Non-Hispanic White	1841	1464 (80.5%)	377 (19.5%)		
	Non-Hispanic Black	1,172	1,029 (88.6%)	143 (11.4%)		
	Asian/Other	866	785 (87.3%)	81 (12.7%)		
Obesity	Non-obese (BMI < 30 kg/m²)	3,253	2,823 (85.0%)	430 (15.0%)	8.298 (1, 30)	0.007
	Obese (BMI ≥ 30 kg/m²)	2,199	1,766 (79.7%)	433 (20.3%)		
Smoking	Non-smoker	3,166	2,747 (84.5%)	419 (15.5%)	3.129 (1.657, 49.723)	0.061
	Former smoker	1,254	1,006 (81.2%)	248 (18.8%)		
	Current smoker	1,027	832 (79.8%)	195 (20.2%)		
Sleep duration	Adequate sleep (≥ 7 hours daily)	4,180	3,528 (83.2%)	652 (16.8%)	1.944 (1, 30)	0.174
	Sleep deprived (< 7 hours daily)	1,238	1,041 (81.9%)	197 (18.1%)		
Depression	Not depressed (PHQ-9 < 10)	2,618	2,283 (84.8%)	335 (15.2%)	7.703 (1, 30)	0.009
	Depressed (PHQ-9 ≥ 10)	169	124 (73.3%)	45 (26.7%)		

The weighted prevalence of tinnitus was 15.0% (*n* = 430; 95% CI: 13.1-17.1) among non-obese adults and 20.3% (*n* = 433; 95% CI: 17.2-23.8) among obese adults (*P* = 0.007), demonstrating a significant positive association between obesity and tinnitus prevalence at the population level (Table 2).

**Table 2:** Weighted prevalence of tinnitus by obesity status. *P*-values are based on the Rao-Scott chi-square test converted to an *F*-statistic to account for the complex survey design of NHANES. CI, confidence interval; df1, numerator degrees of freedom; df2, denominator degrees of freedom; NHANES, National Health and Nutrition Examination Survey

Obesity status	Weighted prevalence of tinnitus, *n* (%)	95% CI	Rao-Scott adjusted *F* (df1, df2)	*P*-value
Non-obese	430 (15.0%)	13.1-17.1	8.298 (1, 30)	0.007
Obese	433 (20.3%)	17.2-23.8		

In the unadjusted logistic regression model, obesity was associated with 44% higher odds of tinnitus (OR = 1.44; 95% CI: 1.11-1.87; *P* = 0.007). After adjustment for age, sex, and race/ethnicity, the association remained significant (OR = 1.40; 95% CI: 1.08-1.82; *P* = 0.013). Further adjustment for smoking status, sleep duration, and depression only modestly attenuated the relationship (OR = 1.41; 95% CI: 1.01-1.98; *P* = 0.044) (Table 3).

**Table 3:** Association between obesity and tinnitus. Adjusted Wald *F*-statistics and *P*-values are from the Complex Samples Logistic Regression accounting for strata, primary sampling units, and sampling weights. CI, confidence interval; df1, numerator degrees of freedom; df2, denominator degrees of freedom

Model	Adjustment	Odds ratio (95% CI) for obesity	Adjusted Wald *F* (df1, df2)	*P*-value
Model 1	Unadjusted	1.44 (1.11-1.87)	8.245 (1, 30)	0.007
Model 2	Age, sex, and race adjusted	1.40 (1.08-1.82)	6.984 (1, 30)	0.013
Model 3	Fully adjusted (age, sex, race, smoking, sleep, depression)	1.41 (1.01-1.98)	4.442 (1, 30)	0.044

These findings indicate that obesity is independently associated with greater odds of tinnitus among US adults, even after accounting for demographic and behavioral factors.

## Discussion

This nationally representative, cross-sectional analysis using NHANES 2015-2018 data demonstrated a significant association between obesity and tinnitus prevalence. The association between obesity and tinnitus persisted even after adjusting for demographic, behavioral, and psychological factors in a nationally representative US sample. Consistent with previously published population-based studies that report tinnitus prevalence between 10% and 20%, the weighted prevalence of self-reported tinnitus was 17.2% (*n* = 863; 95% CI: 15.5-19.0) [12]. A higher prevalence of tinnitus was reported in obese participants (433, 20.3%) as compared to non-obese participants (430, 15%). After accounting for complex survey design, this difference in the odds of self-reported tinnitus between obese and non-obese individuals remained statistically significant.

Our findings of the unadjusted model showed that obesity was linked to higher odds of reporting tinnitus. The association showed a slight attenuation after adjusting for demographic factors (race, sex, and age), but it was still significantly positive. Even after controlling for behavioral and psychosocial factors such as depression, smoking, and lack of sleep, there was still a positive correlation between obesity and tinnitus. This shows that obesity may contribute to tinnitus on its own, but part of the association may also be linked to other conditions such as inflammation, metabolic syndrome, or mental health issues [13].

The observed association between obesity and tinnitus may be explained by several biological mechanisms [14]. Chronic low-grade inflammation, endothelial dysfunction, and vascular compromise, all of which have been linked to obesity, may be associated with alterations in normal cochlear microcirculation and auditory function [15]. Truncal obesity, due to a higher association with cytokine production and pro-inflammatory activity, is believed to significantly influence the development of tinnitus [16,17]. Additionally, metabolic syndrome and insulin resistance associated with obesity may serve as a potential cause of inner ear homeostasis disruption [18]. These changes in response to pathological phenomena may predispose the person to auditory disturbances, including tinnitus. Furthermore, obese individuals are relatively predisposed to sleep disorders and oxidative stress, which may lead to elevated stress hormone levels and ultimately exacerbation of tinnitus perception and distress [19].

Our findings were consistent with previous epidemiological studies that have identified metabolic and vascular risk factors such as dyslipidemia, hypertension, and diabetes as correlates of tinnitus [12]. However, only a few studies specifically evaluate obesity as an independent predictor of tinnitus [20]. The persistent association between exposure and outcome in our study, despite adjusting for multiple variables, underscores the importance of considering body weight and metabolic health in tinnitus research and clinical management. Further studies should incorporate hearing assessments to better clarify whether the observed association reflects otologic, neurologic, or metabolic pathways.

Limitations

Due to its cross-sectional nature, this study reflects an association between obesity and tinnitus but can not establish a causal relationship between the two factors. Self-reported tinnitus was used as the dependent variable, which may be subject to recall bias or misclassification. Additionally, residual confounders such as hearing loss, occupational noise exposure, medication use, or comorbid metabolic conditions may remain accounted for. Hearing loss data may mediate part of the association between obesity and tinnitus, but we focused on the direct relationship independent of auditory thresholds. It is a potential mediator of tinnitus, and without its inclusion, the underlying mechanism linking obesity to tinnitus cannot be fully studied. This omission was due to the incomplete and inconsistent availability of audiometric data across both NHANES cycles. 

## Conclusions

Obesity holds a significant association with self-reported tinnitus in the US population, independent of demographic, behavioral, and psychosocial factors. These findings underscore the potential role of body weight and metabolic health in the development or progression of tinnitus. The substantial burden of obesity in recent years and its association with tinnitus highlight the importance of considering modifiable metabolic risk factors as part of comprehensive tinnitus management. Further research, particularly with the inclusion of audiometric data, is required to understand the pathways linking obesity and tinnitus and determine whether weight reduction might help mitigate tinnitus in the affected population.
